# A Human–Machine Interface Based on Eye Tracking for Controlling and Monitoring a Smart Home Using the Internet of Things

**DOI:** 10.3390/s19040859

**Published:** 2019-02-19

**Authors:** Alexandre Bissoli, Daniel Lavino-Junior, Mariana Sime, Lucas Encarnação, Teodiano Bastos-Filho

**Affiliations:** 1 Postgraduate Program in Electrical Engineering, Federal University of Espirito Santo (UFES), Vitoria 29075-910, Brazil; lucas@ele.ufes.br (L.E.); teodiano.bastos@ufes.br (T.B.-F.); 2 Electrical Engineering Department, Federal University of Espirito Santo (UFES), Vitoria 29075-910, Brazil; daniel_lavino@hotmail.com; 3 Postgraduate Program in Biotechnology, Federal University of Espirito Santo (UFES), Vitoria 29047-105, Brazil; mariana.midori@gmail.com

**Keywords:** human–machine interface (HMI), human–computer interaction (HCI), smart home, eye tracking, assistive technology, usability evaluation, user-centered design (UCD), home automation, Internet of Things (IoT)

## Abstract

People with severe disabilities may have difficulties when interacting with their home devices due to the limitations inherent to their disability. Simple home activities may even be impossible for this group of people. Although much work has been devoted to proposing new assistive technologies to improve the lives of people with disabilities, some studies have found that the abandonment of such technologies is quite high. This work presents a new assistive system based on eye tracking for controlling and monitoring a smart home, based on the Internet of Things, which was developed following concepts of user-centered design and usability. With this system, a person with severe disabilities was able to control everyday equipment in her residence, such as lamps, television, fan, and radio. In addition, her caregiver was able to monitor remotely, by Internet, her use of the system in real time. Additionally, the user interface developed here has some functionalities that allowed improving the usability of the system as a whole. The experiments were divided into two steps. In the first step, the assistive system was assembled in an actual home where tests were conducted with 29 participants without disabilities. In the second step, the system was tested with online monitoring for seven days by a person with severe disability (end-user), in her own home, not only to increase convenience and comfort, but also so that the system could be tested where it would in fact be used. At the end of both steps, all the participants answered the System Usability Scale (SUS) questionnaire, which showed that both the group of participants without disabilities and the person with severe disabilities evaluated the assistive system with mean scores of 89.9 and 92.5, respectively.

## 1. Introduction

People with severe disabilities may have difficulties interacting with their home devices due to the limitations inherent to their disability. Simple activities such as turning on or off a lamp, fan, television, or any other equipment independently may even be impossible for this group of people. With technological advances in the field of sensors and actuators, in recent years some researchers have begun to transfer these technologies to improve the quality of life of people with disabilities, increasing their autonomy regarding the control of existing equipment in the environment [1,2,3,4].

Technologies dedicated to improving the lives of people with disabilities are known as assistive technologies. Assistive technology is an area of knowledge with an interdisciplinary characteristic which encompasses products, resources, methodologies, strategies, practices, and services that aim to promote the functionality related to the activity and participation of people with disabilities, inability, or reduced mobility, aiming at their autonomy, independence, quality of life, and social inclusion [5].

Although many works have been devoted to proposing new assistive technologies to improve the lives of people with disabilities [6,7,8,9,10,11,12,13,14,15], some studies have found that the abandonment of such technologies is quite high, reaching a rate of up to 30% [16,17,18,19]. The reasons for abandoning assistive technology are diverse, the most recurrent being that the user does not like the technology; the user is afraid to use the equipment; the user does not believe in the benefit of the device; the technology is not physically fit for the user; the price of the technology is very expensive; the user does not know how to use the equipment correctly; or the user disapproves of the equipment aesthetic factors [16,17,18,19].

Based on these facts, to avoid or at least reduce the abandonment of new technologies, in developing a new system, engineers should be concerned with developing a system that is useful to the user, i.e., that brings benefits; developing a system to suit the needs of the user; designing tests to validate the technology; evaluating the usability of the system; performing end-user testing; and testing the system outside the laboratory, i.e., testing the system where it will be actually used.

In order to increase the usability of an assistive system, it is also critical to consider the role of human–computer interaction (HCI). The concept of HCI refers to a discipline which studies information exchange between people and computers by using software. HCI mainly focuses on designing, assessing, and implementing interactive technological devices that cover the largest possible number of uses [20]. 

The ultimate goal of HCI is to make this interaction as efficient as possible, looking to minimize errors, increase satisfaction, lessen frustration, include users in development processes, work in multidisciplinary teams, and perform usability tests. In short, the goal is to make interaction between people and computers more productive [21]. 

New technologies have arisen with health-related developments which, by using HCI, meet the needs of different groups such as people with disabilities, the elderly, etc. [22,23]. Although these advances were unthinkable just a few years ago, they are gradually becoming a part of people’s daily lives [24,25].

Human–computer interaction and the need for a suitable user interface has been an important issue in modern life. Nowadays, products and technologies used by society have created concerns about computer technology. For this reason, researchers and designers are interested in the assessment of human and machine behavior, where the machines varying according to the system functionality and the system or product requirements [26].

This work aims to assist people with physical disability to pursue daily living autonomously, taking into account concepts of user-centered design and usability, in order to avoid the abandonment of the proposed system. To this end, we present a new useful assistive system based on eye tracking for controlling and monitoring a smart home, based on the Internet of Things. With this assistive system, a person with severe disabilities was able to control everyday equipment in her residence, such as lamps, television, fan, and radio, and the caregiver was able to remotely monitor the use of the system by the user in real time. In addition, the user interface developed here has some functionalities that allowed improving the usability of the system as a whole.

The subsequent sections of this work are organized as follows. We firstly review the related work and cover some smart homes from around the world in Section 2. In Section 3, we introduce our assistive system and its detailed design. Tests protocols, experimental results, and evaluations are reported in Section 4. Finally, we draw conclusions from our work in Section 5.

## 2. Related Work

In this section, we introduce the state-of-the-art related works by dividing the literature into three parts: (i) user-centered design and usability; (ii) eye tracking; and (iii) smart homes.

### 2.1. User-Centered Design (UCD)

A User-Centered Design (UCD) approach can be used in any type of product from the perspective of HCI design. UCD, also called Human-Centered Design (HCD), is a method that defines the needs, desires, limitations, services, or processes that serve the end-user of a product/system at all stages of a project. In other words, UCD is a multistage troubleshooting process that follows all product development requirements. UCD tries to optimize how the user can use the product/system, what they want or what they need, instead of changing the user’s behavior with the product/system [27].

The approach of UCD is to put human needs, resources, and behavior first, and then design technology to accommodate those needs, resources, and behaviors. It is necessary to understand the psychology and technology to start the design, which requires good communication, mainly between human and machine, indicating available options, the actual status, and the next step [28].

The term “interaction” from human–computer interaction (HCI) is a basis for designing or developing a user interface and an interaction between humans and machines. Preece et al. [29] define four basic activities of an interaction design: (i) identify needs and establish requirements; (ii) develop alternative projects; (iii) construct interactive versions of projects; and (iv) evaluate projects. They also describe three characteristics for interaction design: (i) focus on users; (ii) specific usability criteria; and (iii) iteration. 

Regarding the user experience, Goodman et al. [30] claim that the process is not only to learn about the user experience with the technology, but also for designers to experience interacting in their own work. They report that user experience tests must be applied during the design, the approaches of which could be (i) reported approaches; (ii) anecdotal descriptions; and (iii) first-person research. In addition, Begum [31] presents the user interface (UI), proposing an extended UCD process that adds the “Understand” phase to the methods. The conventional steps of a UCD approach are (i) study, (ii) design, (iii) build, and (iv) evaluate; however, Begum [31] has extended it to (i) understand, (ii) study, (iii) design, (iv) construct, and (v) evaluate.

#### 2.1.1. Usability

The definition of usability is when a product is used by specific users to achieve specific goals with effectiveness, efficiency, and satisfaction in a specific context of use [32]. Usability is more than just about whether users can perform tasks easily; it is also concerned with user satisfaction, where users will consider whether the product is engaging and aesthetically pleasing.

Usability testing is a technique in UCD which is used to evaluate a product by testing it with actual users. It allows developers to obtain direct feedback on how users interact with a product. Thus, through usability testing, it is possible to measure how well users perform against a reference and note if they meet predefined goals, also taking into account that users can do unexpected things during a test. Therefore, to create a design that works, it is helpful for developers to evaluate its Usability, i.e., to see what people do when they interact with a product [26,30].

Usability is then the outcome of a UCD process, which is a process that examines how and why a user will adopt a product and seeks to evaluate that use. That process is an iterative one and seeks to improve the design following each evaluation cycle continuously.

#### 2.1.2. System Usability Scale (SUS)

The System Usability Scale (SUS) provides a reliable tool for measuring usability. It consists of a 10-item questionnaire with five response options which are scored by a 5-point Likert scale, ranging from “1—strongly disagree” to “5—strongly agree”. Originally created by Brooke [33], it allows researchers, engineers, and designers to evaluate a wide variety of products and services, including hardware, software, mobile devices, websites, and applications.

The 10 statements on the SUS are as follows:

(1) I think that I would like to use this system frequently.
(2) I found the system unnecessarily complex.
(3) I thought the system was easy to use.
(4) I think that I would need the support of a technical person to be able to use this system.
(5) I found the various functions in this system were well integrated.
(6) I thought there was too much inconsistency in this system.
(7) I would imagine that most people would learn to use this system very quickly.
(8) I found the system very cumbersome to use.
(9) I felt very confident using the system.
(10) I needed to learn a lot of things before I could get going with this system.

After completion of the questionnaires by the interviewees, the SUS score is calculated as follows:

- For odd-numbered items: subtract 1 from the user response;
- For even-numbered items: subtract the user responses from 5;
- This scales all values from 0 to 4 (with 4 being the most positive response).
- Add the converted responses for each user and multiply that total by 2.5. This converts the range of possible values from 0 to 100 instead of from 0 to 40.

Although the scores are from 0 to 100, these are not percentages and should be considered only in terms of their percentile ranking. Based on the research of Brooke [33], an SUS score above 68 would be considered “above average”, and anything below 68 is “below average”. However, the best way to interpret the results involves normalizing the scores to produce a percentile ranking. This process is similar to grading on a curve based on the distribution of all scores. To get an A (the top 10% of scores), a product needs to score above an 80.3. This is also the score in which users are more likely to be recommending the product to a friend. Scoring at the mean score of 68 gives the product a C, and anything below 51 is an F (putting the product in the bottom 15%).

### 2.2. Eye Tracking

Eye tracking is a technique to measure either the point of gaze (where someone is gazing) or the motion of an eye relative to the head. Eye tracking technology has applications in industry and research in visual systems [34,35,36,37], psychology [38,39], assistive technologies [40,41,42], marketing [43], as an input device for human–computer interaction [44,45,46,47], and in product and website design [48].

Generally, eye tracking measures the eyeball position and determines the gaze direction of a person. The eye movements can be tracked using different methods which can be categorized into four categories: (i) infrared oculography (IROG); (ii) scleral search coil (SSC); (iii) electro-oculography (EOG); and (iv) video-oculography (VOG). SSC measures the movement of a coil attached to the eye [24,49]; VOG/IROG carries out optical tracking without direct contact to the eye [42,47]; and EOG measures the electric potentials using electrodes placed around the eyes [50]. Currently, most of the eye tracking research for HCI is based on VOG, as it has minimized the invasiveness to the user to some degree [40].

The eye is one of main human input media, and about 80 to 90 percent of the outside world information is obtained from the human eye [51]. For communication from user to computer, the eye movements can be regarded as a pivotal real-time input medium, which is especially important for people with severe motor disability, who have limited anatomical sites to use to control input devices [52]. 

The research into eye tracking techniques in HCI is mainly focused on incorporating eye movements into the communication with the computer in a convenient and natural way. The most intuitive solution for incorporating eye movements into HCI is the use of an eye tracker directly connected to a manual input source, such as a mouse. By installing an eye tracker and using its *x*, *y* coordinate output stream as a virtual mouse, the movement of the user’s gaze directly causes the mouse cursor to move (eye mouse). In order to provide such appropriate interaction, several eye-tracking-based control systems have been developed, detailed as follows.

Chin et al. [53] proposed a cursor control system for computer users which integrated electromyogram signals from muscles on the face and point-of-gaze coordinates produced by an eye-gaze tracking system as inputs. Although it enabled a reliable click operation, it was slower than the control system that only used eye tracking, and its accuracy was low. Missimer and Betke [54] constructed a system that used the head position to control a mouse cursor and simulate left-click and right-click of the mouse by blinking the left or right eye. This system relied on the position of the user’s head to control the mouse cursor position. However, irregular movement of the user’s head affected the accuracy of the click function. Lupu et al. [41] proposed a communication system for people with disabilities which was based on a special device composed of a webcam mounted on the frame of a pair of glasses for image acquisition and processing. The device detected the eye movement, and the voluntary eye blinking was correlated with a pictogram or keyword selection, reflecting the patient’s needs. The drawback of this system was that the image processing algorithm could not accurately detect the acquired image (low resolution) and was not robust to light intensity. Later, to improve the reliability of the communication system, they proposed an eye tracking mouse system using video glasses and a new robust eye tracking algorithm based on the adaptive binary segmentation threshold of the acquired images [42].

Lately, several similar systems have also been developed [55,56], and the main concept of these systems is to capture images from a camera, either mounted on headgear worn by the user or mounted remotely, and extract the information from different eye features to determine the point of the gaze. Since, at the time the research was performed, commercial eye trackers were prohibitively expensive to use in HCI, all the aforementioned eye tracking control systems were proposed with self-designed hardware and software. It was difficult for these systems to achieve widespread adoption, as the software and hardware designs were closed source.

### 2.3. Smart Homes

There are many motivations to design and develop applications in smart homes, the main ones being independent living [3,7,9,10,11,57]; wellbeing [4,6,8,12,58]; efficient use of electricity [59,60,61,62,63,64,65,66,67,68,69,70,71,72]; and safety and security [73,74,75,76,77,78,79,80,81,82,83,84,85,86,87,88].

The expression “smart home” is used for a home environment with advanced technology that enables control and monitoring for its occupants and boosts independent living through sensors and actuators to control the environment or through wellness forecasting based on behavioral pattern generation and detection. A variety of smart home systems for assisted living environments have been proposed and developed, but there are, in fact, few homes that apply smart technologies. One of the main reasons for this is the complexity and varied design requirements associated with different domains of the home, which are communication [89,90,91,92,93,94,95], control [96,97,98,99,100,101,102,103,104,105,106,107,108,109,110], monitoring [111,112,113,114,115,116], entertainment [117,118,119,120], and residential and living spaces [121,122].

As an important component of the Internet of Things (IoT), smart homes serve users effectively by connecting them with devices based on IoT. Smart home technology based on IoT has changed human life by providing connectivity to everyone regardless of time and place [123,124]. Home automation systems have become increasingly sophisticated in recent years, as these systems provide infrastructure and methods to exchange all types of appliance information and services [125]. A smart home is a domain of IoT, which is the network of physical devices that provides electronic, sensor, software, and network connectivity inside a home.

There are many smart home systems across the world, most notably in Asia, Europe, and North America. In Asia, it is important to highlight Welfare Techno Houses [126,127], Smart House Ikeda [128], Robotics Room and Sensing Room [129], ActiveHome [130], ubiHome [131,132,133,134], Intelligent Sweet Home [135], UKARI Project and Ubiquitous Home [136,137], and Toyota Dream Home PAPI [138]. In Europe, there are comHOME [139], Gloucester Smart Home [140], CUSTODIAN Project [141], Siemens [142], myGEKKO [143], and MATCH [144]. In North America, there are Adaptive Smart House [145,146], Aware Home Research Initiative (AHRI) [147], MavHome Project [148,149], House_n (MIT House) [150,151], EasyLiving Project [152], Gator Tech Smart House [153], DOMUS Laboratory [154], Intelligent Home Project [155], CASAS [156,157], Smart Home Lab [158,159], AgingMO [160], and Home Monitoring at Rochester University [161].

## 3. Proposed Assistive System

**Figure 1:**
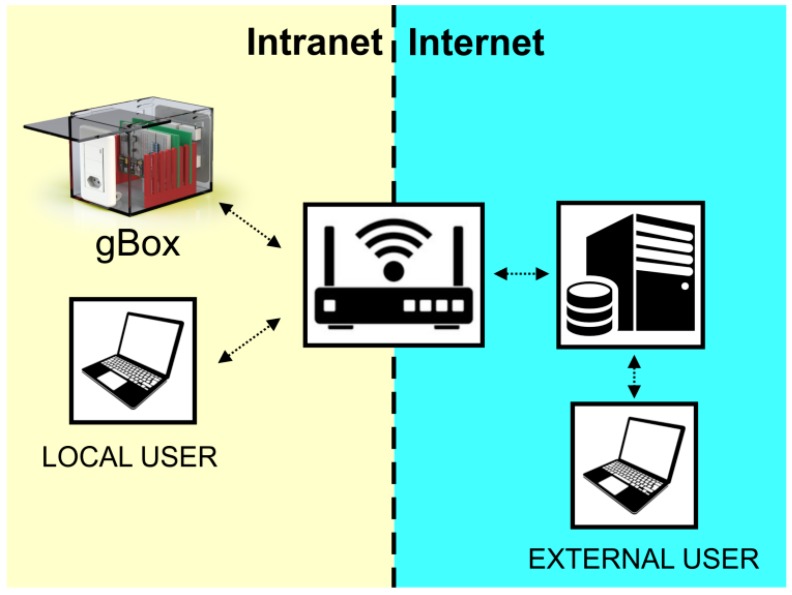
System overview.

The assistive system proposed here empowers people with severe physical disability and mitigates the limitations in everyday life with which they are confronted. The system aims at assisting people with physical disability to pursue daily living autonomously. In Figure 1, the local user is the person with disability who can control the equipment of his/her smart home through eye gaze using the device controller (gBox). At the same time, the caregiver (external user) can monitor the use of the system.

The proposed eye-gaze-tracking-based control system is a software application using a low-cost eye tracker (e.g., The Eye Tribe 1.0 and Tobii Pro). The application detects the user’s gaze with the “mouse cursor control” function provided by the eye tracker. The mouse cursor control allows users to redirect the mouse cursor to the gaze position. Therefore, the system realizes where the user is gazing according to the position of the mouse cursor. By gazing at the point for few seconds, the tool generates the corresponding event. This way, users can select and “click” the corresponding action. The eye tracker detects and tracks the coordinates of the user’s eye gaze on the screen; this made it possible to create applications that can be controlled in this way.

The eye tracker software is based on an open Application Programming Interface (API) that allows applications (clients) to communicate with the eye tracker server to obtain eye gaze coordinates. The communication is based on messages sent asynchronously, via Socket, using the Transmission Control Protocol (TCP).

It should be noted that to use this assistive system, it is not necessary to install any software in addition to the Internet browser, as the web application was developed to run in Internet browsers. It is only recommended to use the most up-to-date versions of well-known browsers, such as Google Chrome (preferable), Mozilla Firefox, or Internet Explorer.

### 3.1. System Architecture

**Figure 2:**
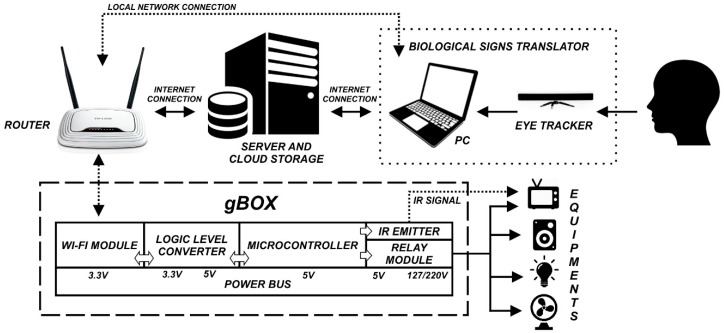
Functional model of the eye/gaze-tracking-based control system.

Different systems for HCIs based on biological signals have been proposed with various techniques and applications [162]. Despite each work presenting unique properties, most of them fit into a common framework. Figure 2 shows the framework adopted in this system.

Observing the model, a loop structure can be recognized; it starts from the User, whose biological signals are the primary input, and ends with the environment that is affected by the actions of the system. Along this path, three main modules can be identified: the Biological Signal Translator module, the Server and Cloud module, and the Device Controller/Device module (gBox). Eventually, the loop is closed through user feedback of different kinds. The communication between the modules is bidirectional in order for a module to know the outcome of a command.

### 3.2. Connectivity

The web application was developed to work both online and offline. To open the online application and work in “online mode”, the user must simply enter the Internet browser and the domain where it is hosted (https://ntagbox.000webhostapp.com). The online application can be hosted on any HTTP server; the domain used in this work is provided free of charge by “Hostinger”, with limited, but sufficient, configurations. Because there is an external server, an Internet connection is necessary. In this case, the connection is via WebSocket (WS), which is the best option, as the connection between the application and the physical device is done over the Internet; in this way, the user commands are stored on the server instantly.

On the other hand, to use the offline application and work in the “offline mode”, it is only necessary to have the site files in a folder on the computer, run the “intex.html” file, and the browser will run the application. In this case, the connection is via AJAX, which is used when there is no Internet access in the user’s home (or if the Internet drops out temporarily); thus, the connection between the application and the physical device is made by the Intranet, and in this way, the user commands are stored temporarily on the computer until the connection via WS is established.

**Figure 3:**
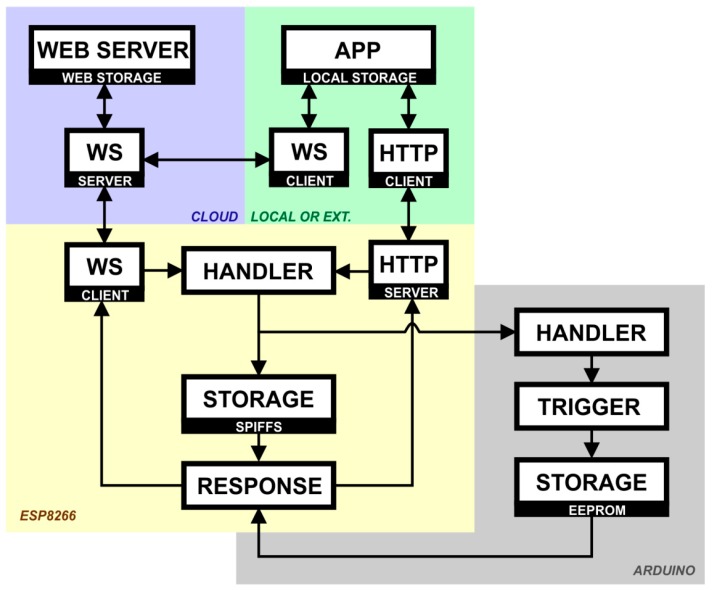
Connectivity of the assistive system.

In order for the local and external connection to be implemented on the physical device, two ways of WEB communication were created (Figure 3): HTTP and WS. Once a command is launched from the application (APP), an internal mechanism identifies whether there is access to the external (Internet) server or not (local connection), and also identifies whether the physical device is properly connected to that server. The application has two communication clients related to the two communication channels. If the device is connected on the Internet, WS is used as the communication channel both in the application and in the device since it is capable of establishing an endless connection with the server, allowing the data to be sent bidirectionally and asynchronously. When there is no Internet connection and the application is in the same local network as the physical device, the communication channel used is HTTP, with an HTTP server on the physical device so that it has an IP that identifies it locally.

In the physical device, the data packets can be received by the two communication channels. However, they are directed to a single channel—the HANDLER—that handles the information contained therein. The HANDLER has the function of authenticating the data received and identifying the command contained therein so that the TRIGGER can be activated. The TRIGGER is the set of triggers and sensors responsible for interacting with the user’s devices. Some commands simply require changes in the internal variables of the system. For this purpose, it also has a small non-volatile SPIFFS memory module responsible for storing such variables, such as SSID and Wi-Fi password; user password; relay states; etc. It is also essential to keep the data stable if there is any power outage. If everything succeeds, the RESPONSE block returns the confirmation message to the application, returning to the input path of the packet.

If this input path is the WS client, the packet will be returned to the WS server in the cloud, which will store the command so that it is accessible via Internet, and finally send the confirmation to the WS client of the application. If this input path is the HTTP server, the device returns the response directly to the application, which places it in a rank to be sent to the server as soon as an Internet connection is established.

### 3.3. GlobalBox (gBox)

**Figure 4:**
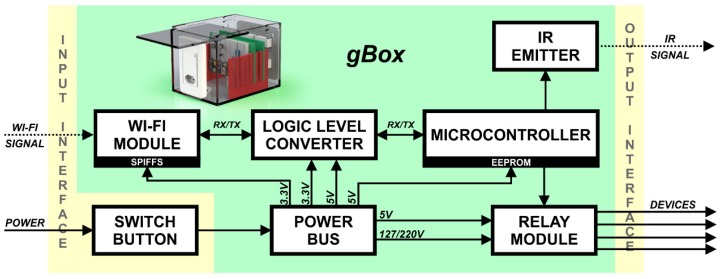
GlobalBox (gBox) of the assistive system.

The GlobalBox (gBox) is the device controller module of the smart home. Figure 4 shows, in a simplified form, the main components of the gBox.

Before starting the application, the switch button must be turned on. With the box powered up, it is possible to receive information through the Wi-Fi module. This information is the command sent by the user through the user interface running on the computer. The information received by the Wi-Fi module is processed in the microcontroller, and then the actions corresponding to the received commands are performed, being able to turn the relays of the equipment on or off or send specific commands by the infrared (IR) emitter to control the functions of the TV or radio.

### 3.4. Wireless Infrared Communication

Infrared (IR) signals are essential to control some residential devices, such as TVs and radios. The gBox has an internal library with a set of IR protocols (the most used) already implemented, which assists in the task of modulation and demodulation of IR signals. The hardware consists of an IR detector that receives signals at 38 kHz, an IR emitter, and the microcontroller, which is responsible for treating and storing the signal in memory for later use.

**Figure 5:**
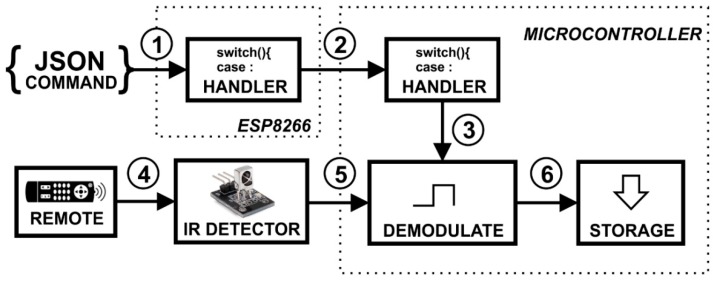
Detection and storage of an IR command.

To store the code of any remote control, it is necessary to send the read command from the application so the demodulator will be activated; then, the caregiver can press the button (towards the IR detector) that he/she wants to record, which transforms the received IR signals into codes that can be stored in memory (Figure 5).

**Figure 6:**
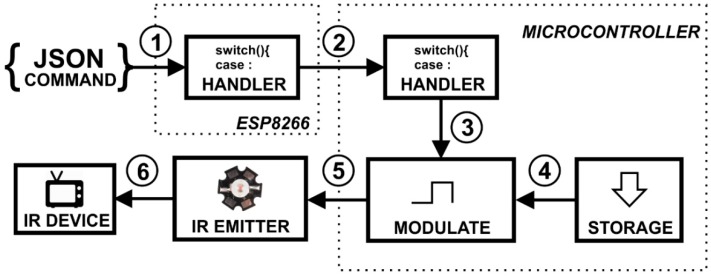
Circuit of IR emission commands previously stored.

To emit an IR signal, it is necessary to start the application whose command activates the signal modulator, which takes the codes stored in the memory of the microcontroller and transforms them into the original IR signal, sending it to the IR emitter module (Figure 6). The emitter module, when pointed towards the target device, acts in the same way as the remote control in its respective function.

### 3.5. User Interface

The most well-known strategy of eye tracking applications is using the gaze to perform tasks of pointing and selecting. However, the direct mapping of the gaze (more specifically, of fixations) to a command of system selection creates a problem, called the “Midas Touch”, in which a selection can be activated in any screen position observed by the user, whether they intended to do it or not.

Thus, after filtering the eye tracker data, the Midas Touch problem must be avoided by implementing mechanisms for the user to indicate when he/she really desires to perform a selected command. The first approach to this problem is to implement a dwell time, in which the selection of one option is done only after a time interval. 

**Figure 7:**
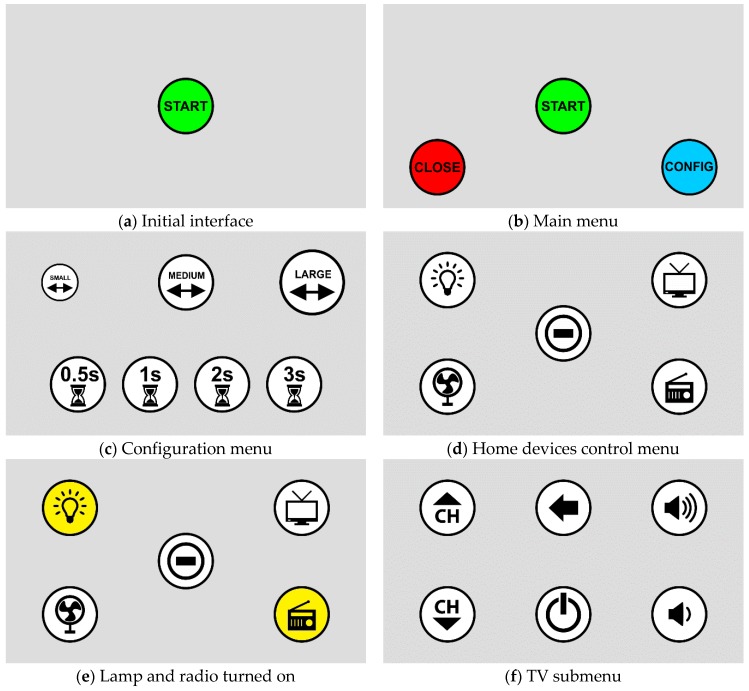
User interface.

Figure 7a shows the initial screen when the system is off. When the user turns the system on by selecting the “Start” icon, the main menu shown in Figure 7b appears. In Figure 7b, the user has three options: (i) “Close”, to close the user interface of the system; (ii) “Start”, to open the home devices control menu; and (iii) “Config”, to open the configuration menu.

It is important for users to be able to turn the system on and off. That is why the interface presented in Figure 7b was included. If the user chooses “Close”, the system is closed and returns to the initial interface.

When the user selects the “Config” option in Figure 7b, the configuration menu shown in Figure 7c appears. In the configuration menu, the user can choose the icon size and the dwell time. There are three options for the icon size: (i) “Small”; (ii) “Medium”; and (iii) “Large”. There are four options for dwell time: (i) 0.5 s; (ii) 1.0 s; (iii) 2.0 s; and (iv) 3.0 s. After choosing any of the options, the interface automatically returns to the main menu with the new configuration saved.

When the user selects the “Start” option in Figure 7b, the control menu of the home devices shown in Figure 7d appears. In this menu, the user is presented with four options of devices to be turned on/off: a fan, TV, lamp, and radio. In addition, there is an option to close that menu to return to the main menu (Figure 7b) by selecting the center icon. The icon size on the interface and dwell time used are the ones that the user selected in the configuration menu. 

When the device is turned on, the background of the icon becomes yellow, like “Lamp” and “Radio” are in Figure 7e. Fan and TV are turned off; thus, the background of the icons is white. After turning the desired device on or off, the user can give the command “Close” and turn the system off. This command closes the interface, but the system keeps the selected devices in their current state (on or off).

After selecting the TV icon, the interface displays the TV submenu shown in Figure 7f. In the TV submenu, the user can turn the TV on or off, change the channel “up” or “down”, increase or decrease the volume, and close the TV submenu. Is this last option, the TV submenu is closed and the interface returns to the home devices control menu, but the system keeps the TV in its current state (on or off).

### 3.6. Caregiver Interface

The gBox Central Management is accessible from the website (https://ntagbox.000webhostapp.com/). In the header of the caregiver interface are the top bar and titles. Before giving any command in the application, it is required to enter the password in the password field of the bar. After that, it is recommended to click “Update Status” so that the application synchronizes with the current states of the equipment. The “Start Application” button opens the user interface so the user can control the smart home with the eye tracker. On the right side of the bar, the connection status between the application and the physical devices is reported. The connection flag is independent of the access password to be updated. There are three possible connection status:

- Connected via WS. This is the best connection. It occurs when the connection between the application and the physical device is done by Internet; that way, user commands are stored on the server instantly.
- Connected via AJAX. This occurs when the connection between the application and the physical device is made by the Intranet, so the commands are stored temporarily on the user’s computer until a connection via WS is established.
- Not Connected. This occurs when there is no connection between the application and the physical device. In this case, it is suggested to refresh the site and check the connections with the physical device.

The body of the application is composed of seven sections: (i) Last Commands; (ii) General Notifications; (iii) Infrared Remote Control Settings; (iv) Remote Actuation; (v) Data Acquisition; (vi) Change Password; and (vii) Change Wi-Fi Password, the details of which are as follows.

(i) “Last Commands”: In this section, the commands successfully performed are presented, as well as the date and time they occurred. To appear in this list, it is necessary to update the states after establishing a WS connection.

(ii) “General Notifications”: In this section, all the notifications from the application features are presented; for example, “updating status” or “the status are updated”.

(iii) “Infrared Remote Control Settings”: In this section, it is possible to update the IR commands by pressing the “READ” button and follow the instructions displayed in the general notifications section. In addition, hexadecimal codes and protocols of the buttons/commands of the IR control are presented.

(iv) “Remote Actuation”: Remote actuation can be used to control the smart home application by using the caregiver application.

(v) “Data Acquisition”: This section allows the download of the list of commands based on a specified time interval. The downloaded text file can be accessed in any text editor or spreadsheet analysis program.

(vi) “Change Password”: This section allows the change of the user password. It is necessary that the password field of the upper bar be correctly filled with the old password.

(vii) “Change Wi-Fi Password”: This functionality allows the change of the passwords of the SSID and of the Wi-Fi. It is necessary that the password field of the upper bar be correctly filled with the user password.

## 4. Tests, Results, and Discussion

In this section, we report the experiments, which were divided into two steps, and analyze and discuss the results. In the first step, the proposed assistive system was assembled in an actual home where tests were conducted with 29 participants (group of able-bodied participants). In the second step, the system was tested for seven days, with online monitoring, by a person with severe disability (end-user) in her own home, not only to increase her convenience and comfort, but also so that the system would be tested where it would in fact be used. The objective of this test was to explore the effectiveness of the assistive system, the ability of the participant to learn how to use the system, and the efficiency and the usability of the proposed user interface.

According to Resolution No. 466/12 of the National Health Council of Brazil, the research was approved by the Committee of Ethics in Human Beings Research of the Federal University of Espirito Santo (CEP/UFES) through opinion nº 2.020.868, of April 18, 2017.

### 4.1. Tests with a Group of Able-Bodied Participants

#### 4.1.1. Pre-Test Preparation

Initially, we fully installed the system and tested all possible commands to verify that the system was working properly. Some errors were found and quickly corrected so that the system was considered to work perfectly before we started the tests with the participants.

Afterwards, a pilot test was conducted with one of the involved researchers to rehearse the procedure before conducting the study with the participants. The researcher completed all the data collection instruments. The problems encountered during the pilot test helped to identify changes before conducting the experiment with the participants.

#### 4.1.2. Participants

To test the system, 29 healthy subjects (group of able-bodied participants) participated in the research with the assistive system in the smart home. The participants were 18 men and 11 women, all aged from 17 to 40 (average: 28) and height from 1.50 to 1.94 (average: 1.71). Some of the participants had had at least one experience with HCI through biological signals, but almost none of them had used eye tracking.

Of the total, 12 participants (#2, #8, #9, #11, #12, #14, #15, #16, #17, #22, #25, and #26) wear glasses; however, all of them performed the test without their glasses. In some cases, it was by preference of the participant himself/herself, but in most of the cases, their glasses had anti-reflective film which prevented, or at least disturbed, the infrared eye tracker passing through the lens of the glasses to obtain the correct position of the eyes of the participant. This is an important limitation of this study.

#### 4.1.3. Experimental Sessions

The tests started by welcoming the participants and making them feel at ease. The participants were given an overview of the system and the test and were told that all their information will be kept private. 

**Figure 8:**
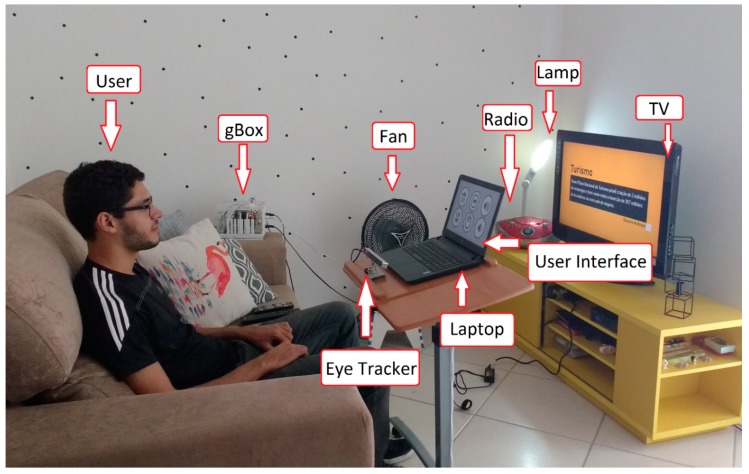
Able-bodied participant testing the system in the smart home.

Each participant was seated on a couch in front of the laptop that contained the user interface, and the eye tracker was positioned properly pointing to his/her eyes (Figure 8).

After that, the participant performed the calibration stage of the eye tracker, following the manufacturer’s guidelines. Each participant performed the test over about five to ten minutes. It was required of the participants to use the system long enough so they could test all the functionality options available.

It is important to mention that the user was positioned facing a glass door that provided access to a balcony, with high incidence of sunlight. Despite this, the sunlight did not cause a problem in performing the experiments, showing the robustness of the eye tracker.

It can be seen in Figure 8 that the eye tracker was positioned towards the user’s eyes. In the course of the experiments, it is normal for a person to move his/her head a little, moving away from the location where the eye tracker was calibrated. We note that small vertical and horizontal position variations (around 5 to 10 cm) did not significantly interfere with the system performance. However, if the user’s eyes are completely out of range of the eye tracker, then he/she will not be able to send commands to the system. In this case, it may be necessary to reposition the eye tracker and calibrate it again. Considering that this system was designed to be used by people with severe disabilities, it is not expected that they will have wide head movements. 

After the end of the session, the participant answered the SUS questionnaire and was encouraged to make suggestions.

#### 4.1.4. Results and Discussion

Of the able-bodied participants, 3 opted for the small interface icon size option, 22 opted for medium, and 4 opted for large. As predicted in our previous work [163], most users opted for the medium size option. However, it is important to note that seven participants (24% of the total) preferred another size, thus showing the advantage of having options available.

For dwell time, 6 participants opted for 0.5 s, 18 opted for 1.0 s, 5 opted for 2.0 s, and no one chose the option of 3.0 s. Again, as predicted in our previous work [163], most users opted for the option of 1.0 s. However, it is important to note that 11 participants (38% of the total) preferred another time interval, thus showing the advantage of having this functionality available.

In fact, only 14 participants opted for the combination of medium icon size and 1.0 s dwell time. In other words, 15 participants (52% of the total) preferred another size or other time interval, and this shows the importance of having options to choose from in order to increase the usability of the system.

**Figure 9:**
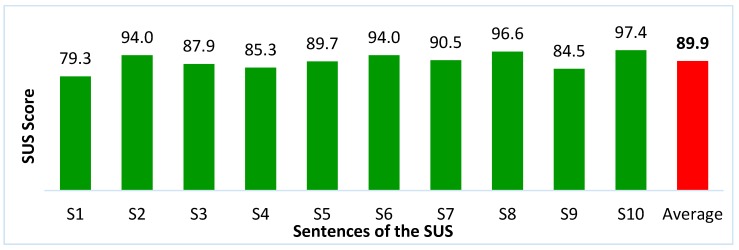
System Usability Scale (SUS) score of each of the ten items of the SUS.

Regarding the usability, three participants gave a maximum SUS score, and the lowest result was 75. Thus, the overall mean was 89.9, with a standard deviation of 7.1. It is worth mentioning that products evaluated above 80.3 are in the top 10% of the scores. In fact, according to Brooke [33] and Bangor [164], products evaluated in the 90 point range are considered exceptional, products evaluated in the range of 80 points are considered good, and products evaluated in the range of 70 points are acceptable. Figure 9 presents the SUS score of each item evaluated by the participants regarding the assistive system.

The items regarding the available functionality, the complexity in using, and the confidence in functioning all received scores above 80. The lowest score obtained (79.3) was related to the sentence S1, which is about interest in using the system frequently. Many participants said that they would not have much interest in using this system, as the system was designed for a person with severe disability, which is not the case for the participant (able-bodied). This reinforced the need to test the system with people with severe disabilities.

### 4.2. Tests with a Person with Disabilities

#### 4.2.1. Pre-Test Preparation

Initially, an interview was conducted by the occupational therapist of our research group to better understand the potential participant. At this point, relevant information was gathered about her disability and daily life, whether there was interest in participating in the study, in what activities she was most involved, and what tasks she would like to be able to do or have the assistance of the technology to execute.

After this first contact, the information was brought to the research group and the candidate was selected to participate in the experiments. A telephone appointment was made between the occupational therapist and the participant’s husband at their home, where the system would be used.

#### 4.2.2. Participant Background

The participant is female and 38 years old. She was diagnosed in June 2012 with Wernicke’s Encephalopathy. Thus, the participant presents a lack of coordination of movements (ataxia) and extreme difficulty in balancing and walking. Her most difficult activities are those that require manual dexterity, such as typing on the computer, writing, using a mobile device, and handling the TV remote control. In addition, the participant has difficulty walking, considered practically impossible by her, or when necessary, causing enormous discomfort.

#### 4.2.3. Experimental Sessions

**Figure 10:**
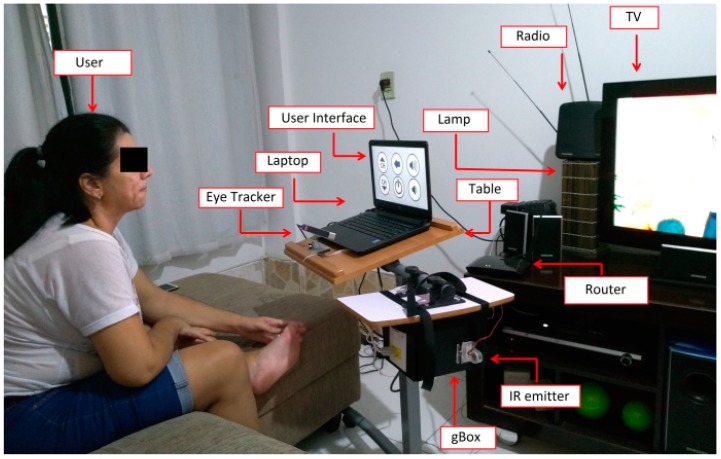
Participant with disabilities testing the system at her home.

To test the assistive system, it was firstly fully assembled and configured in the home of the end-user, who agreed to participate in the experiments (Figure 10). The participant was given an overview of the system and test. Before proceeding to the test, one of the researchers performed all possible commands to verify that the system was working properly.

The participant was seated on a couch in front of the laptop that contained the user interface, and the eye tracker was positioned properly, pointing towards her eyes (Figure 10). After that, the participant performed the calibration stage of the eye tracker, following the manufacturer’s guidelines. The participant performed the test over seven days. It was required of the participant to use the system long enough so she could test all the functionality options available.

After the end of the session, the participant answered the SUS questionnaire and was also encouraged to make suggestions.

#### 4.2.4. Results and Discussion

According to information obtained in the interview with the participant, Friday and Saturday were the best days of the week for her to receive the researchers in her home, so she opted to install the system on a Friday (09/14/2018) and uninstall it on a Saturday (10/06/2018). Before the experiments, the participant informed us that she was not able to use the equipment on Sundays and Mondays, as on Sunday she usually receives many relatives in her house, and on Monday she spends the whole day away from home. In addition, the participant informed that she would need to make a trip for personal reasons during the experiment (from 09/23/2018 until 10/01/2018). All these situations put forward by the participant were considered pertinent, as they actually depict the daily life of a person with disability, revealing how technology needs to adapt to the person’s life. In addition, it was considered interesting to evaluate if the participant would use the system after she was away from home for a few days without using it. In many cases, assistive technology is abandoned, which did not happen with our system.

Table 1 shows the use of the assistive system by the participant, which shows the number of hours the system was used during the seven days of use.

It is important to note that the system was not only used, but used for several hours over several days, which was considered better than expected. The tests previously performed with the group of able-bodied participants of only 5 to 10 minutes—although important for evaluating the system with several users—were much less representative than the present test with the person with disabilities, which had a total duration of more than 20 hours. Another fact that corroborates this is the number of commands performed by the system over the days, shown in Table 1. Note that the system received a total of 542 commands and, as reported by the user, worked perfectly.

To better understand how the system was used by the participant, Table 2 summarizes the complete information about the commands received by the system throughout the complete test. The system was always used between 2:00 p.m. and 10:00 p.m., and more than 80% of the commands were sent between 1:00 p.m. and 4:00 p.m., indicating a user routine.

Regarding the usability (SUS), the user gave the maximum score for all the questions regarding the willingness to use the system, available functions, ease and confidence in using it, etc. So, focusing on the only feature that the user rated low, according to her, she needed to learn many new things to be able to use the system and so she gave a low score on that item. She believes that after using the system more, she will not need to learn so much more additional information.

Despite this, the user evaluated the system with an average of 92.5, which is quite high, even higher than the previous tests with the group of able-bodied participants, in which the overall mean was 89.9. In fact, according to Brooke [33] and Bangor [164], products evaluated in the 90 point range are considered exceptional.

As recommended by Begum [31], in this work the methodology of UCD for the design of new products was used in order to put the needs and desires of the user first. This way, it was possible to understand, study, design, build, and evaluate the system from the user’s point of view.

## 5. Conclusions

This work presented an assistive system, based on eye gaze tracking for controlling and monitoring a smart home using the Internet of Things, which was developed following concepts of user-centered design and usability. The proposed system allowed a user with disabilities to control everyday equipment in her residence (lamps, television, fan, and radio). In addition, the system could allow the caregiver to remotely monitor the use of the system by the user in real time. The user interface developed included some functionality to improve the usability of the system as a whole. The experiments were divided into two steps. In the first step, the assistive system was assembled in an actual home where tests were conducted with 29 participants (group of able-bodied participants). In the second step, the system was tested for seven days, with online monitoring, by a person with disability (end-user). The results of the SUS showed that the group of able-bodied participants and the end-user evaluated the assistive system with mean scores of 89.9 and 92.5, respectively, positioning the tool as exceptional.

## Figures and Tables

**Table 1:** Summary of system usage information.

Date	Start	End	Duration	Commands
09/14/2018	14:20	18:10	03:50:00	163
09/28/2018	12:51	19:49	06:58:00	131
09/20/2018	13:58	15:55	01:57:00	36
09/22/2018	15:52	17:36	01:44:00	18
10/02/2018	17:38	18:01	00:23:00	31
10/04/2018	14:15	18:04	03:49:00	88
10/05/2018	14:35	15:56	01:21:00	75
**Total**			**20:02:00**	**542**

**Table 2:** Hourly distribution of the commands throughout the days of use of the assistive system.

From	To	Day 1	Day 2	Day 3	Day 4	Day 5	Day 6	Day 7	Percentage
00	12	0	1	0	0	0	0	0	0%
12	13	0	1	0	0	0	0	0	0%
13	14	0	62	7	0	0	0	0	13%
14	15	94	7	22	0	0	75	31	42%
15	16	57	21	7	13	0	3	44	27%
16	17	0	6	0	1	0	0	0	1%
17	18	0	0	0	4	22	8	0	6%
18	19	12	3	0	0	5	0	0	4%
19	20	0	31	0	0	0	0	0	6%
20	21	0	0	0	0	0	0	0	0%
21	22	0	0	0	0	4	2	0	1%
22	00	0	0	0	0	0	0	0	0%
**Total**		**163**	**131**	**36**	**18**	**31**	**88**	**75**	**542**

## Abbreviations

The following abbreviations are used in this manuscript:

AJAX	Asynchronous JavaScript and XML
API	Application Programming Interface
CEP/UFES	Committee of Ethics in Human Beings Research of the Federal University of Espirito Santo
EEPROM	Electrically-Erasable Programmable Read-Only Memory
EOG	Electrooculography
HCI	Human-Computer Interaction
HMI	Human-Computer Interface
HTTP	Hypertext Transfer Protocol
IoT	Internet of Things
IP	Internet Protocol
IR	Infrared
IROG	Infrared Oculography
JSON	JavaScript Object Notation
SPIFFS	Serial Peripheral Interface Flash File System
SSC	Scleral Search Coil
SSID	Service Set IDentifier
SUS	System Usability Scale
TCP	Transmission Control Protocol
UCD	User-Centered Design
UI	User Interface
VOG	Video-Oculography
WS	WebSocket
